# Methyl 2-(4-hy­droxy­benzo­yl)benzoate

**DOI:** 10.1107/S1600536811026651

**Published:** 2011-07-09

**Authors:** M. S. Siddegowda, Jerry P. Jasinski, James A. Golen, H. S. Yathirajan, M. T. Swamy

**Affiliations:** a Department of Studies in Chemistry, University of Mysore, Manasagangotri, Mysore 570 006, India; b Department of Chemistry, Keene State College, 229 Main Street, Keene, NH 03435-2001, USA; c Department of Chemistry, Sambhram Institute of Technology, Bengaluru, India

## Abstract

In the title compound, C_15_H_12_O_4_, the dihedral angle between the benzene rings is 64.0 (6)°. In the crystal, mol­ecules are linked by O—H⋯O hydrogen bonds, forming *C*(8) chains propagating in [10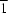] and the packing is reinforced by weak C—H⋯O inter­actions.

## Related literature

For background to benzophenone derivatives, see: Sieroń *et al.* (2004) ▶. For related structures, see: Cox *et al.* (2008 ▶); Jasinski *et al.* (2009 ▶). For reference bond lengths, see: Allen *et al.* (1987 ▶).
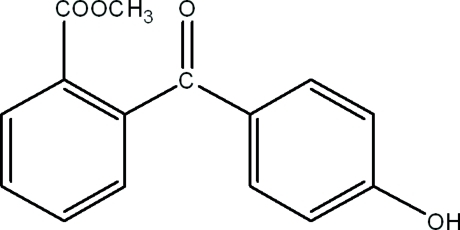


## Experimental

### 

#### Crystal data

C_15_H_12_O_4_
*M* *_r_* = 256.25
Monoclinic, 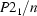

*a* = 8.9017 (12) Å
*b* = 13.9940 (17) Å
*c* = 10.0473 (12) Å
β = 94.687 (12)°
*V* = 1247.4 (3) Å^3^
*Z* = 4
Mo *K*α radiation
μ = 0.10 mm^−1^
*T* = 173 K
0.38 × 0.32 × 0.24 mm

#### Data collection

Oxford Diffraction Xcalibur Eos Gemini diffractometer
Absorption correction: multi-scan (*CrysAlis RED*; Oxford Diffraction, 2010 ▶) *T*_min_ = 0.963, *T*_max_ = 0.977
11331 measured reflections
3231 independent reflections
2722 reflections with *I* > 2σ(*I*)
*R*_int_ = 0.020

#### Refinement

*R*[*F* ^2^ > 2σ(*F* ^2^)] = 0.042
*wR*(*F* ^2^) = 0.117
*S* = 1.02
3231 reflections
177 parameters
1 restraint
H atoms treated by a mixture of independent and constrained refinement
Δρ_max_ = 0.25 e Å^−3^
Δρ_min_ = −0.18 e Å^−3^

### 

Data collection: *CrysAlis PRO* (Oxford Diffraction, 2010 ▶); cell refinement: *CrysAlis PRO*; data reduction: *CrysAlis RED* (Oxford Diffraction, 2010 ▶); program(s) used to solve structure: *SHELXS97* (Sheldrick, 2008 ▶); program(s) used to refine structure: *SHELXL97* (Sheldrick, 2008 ▶); molecular graphics: *SHELXTL* (Sheldrick, 2008 ▶); software used to prepare material for publication: *SHELXTL*.

## Supplementary Material

Crystal structure: contains datablock(s) global, I. DOI: 10.1107/S1600536811026651/hb5942sup1.cif

Structure factors: contains datablock(s) I. DOI: 10.1107/S1600536811026651/hb5942Isup2.hkl

Supplementary material file. DOI: 10.1107/S1600536811026651/hb5942Isup3.cml

Additional supplementary materials:  crystallographic information; 3D view; checkCIF report

## Figures and Tables

**Table 1:** Hydrogen-bond geometry (Å, °)

*D*—H⋯*A*	*D*—H	H⋯*A*	*D*⋯*A*	*D*—H⋯*A*
O4—H4*O*⋯O3^i^	0.85 (1)	1.88 (2)	2.7310 (14)	173 (2)
C12—H12*A*⋯O2^i^	0.95	2.50	3.4333 (15)	167

Symmetry code: (i) 
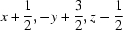
.

**Fig. 1.**
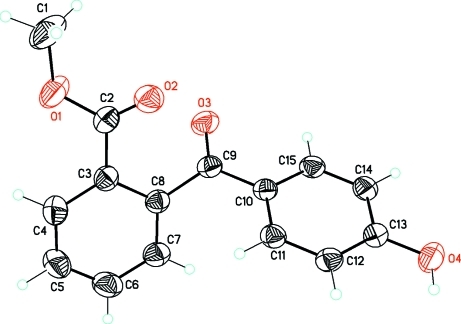
Molecular structure of the title compound showing 50% probability displacement ellipsoids.

**Fig. 2.**
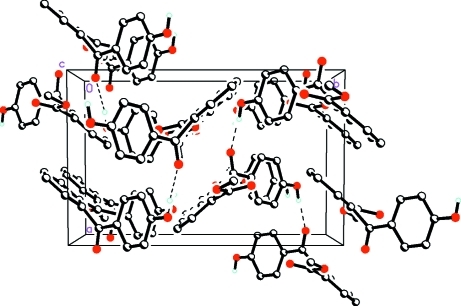
Packing diagram of the title compound viewed down the c axis. Dashed lines represent O—H···O hydrogen bonds.

## supplementary crystallographic information

### Comment

The title compound is a starting material for the synthesis of pitofenone, which is an antispasmodic. More generally, benzophenone derivatives have many applications in organic chemistry (Sieroń *et al.*, 2004). The crystal structures of some substituted benzophenones (Cox *et al.*, 2008) and 2-amino-5-nitrophenyl 2-chlorophenyl ketone (Jasinski *et al.*, 2009) have been reported.  In view of the importance of the title compound, this paper reports the crystal structure of (I), C_15_H_12_O_4_.

In the title compound, C_15_H_12_O_4_, the dihedral angle between the mean planes of the two benzene rings is 64.0 (6)° (Fig. 1).  Bond distances are in normal ranges (Allen *et al.*, 1987).  Crystal packing is stabilized by O—H···O hydrogen bonds and weak C—H···O intermolecular interactions (Table 1, Fig. 2).

### Experimental

The title compound was obtained as a gift sample from R. L. Fine Chem, Bengaluru, india.  Colourless blocks were grown from methanol solution (430-433 K).

### Refinement

The O–H atom was located by Fourier analysis and refined isotropically with DFIX = 0.84Å. All of the remaining H atoms were placed in their calculated positions and then refined using the riding model with Atom–H lengths of 0.95Å (CH) or 0.98Å (CH_3_).  Isotropic displacement parameters for these atoms were set to 1.18-1.20 (CH) or 1.51 (CH_3_) times *U*_eq_ of the parent atom.

### Crystal data

**Table:**

C_15_H_12_O_4_	*F*(000) = 536
*M**_r_* = 256.25	*D*_x_ = 1.364 Mg m^−^^3^
Monoclinic, *P*2_1_/*n*	Mo *K*α radiation, λ = 0.71073 Å
Hall symbol: -P 2yn	Cell parameters from 4807 reflections
*a* = 8.9017 (12) Å	θ = 3.3–32.3°
*b* = 13.9940 (17) Å	µ = 0.10 mm^−^^1^
*c* = 10.0473 (12) Å	*T* = 173 K
β = 94.687 (12)°	Block, colorless
*V* = 1247.4 (3) Å^3^	0.38 × 0.32 × 0.24 mm
*Z* = 4	

### Data collection

**Table:**

Oxford Diffraction Xcalibur Eos Gemini diffractometer	3231 independent reflections
Radiation source: Enhance (Mo) X-ray Source	2722 reflections with *I* > 2σ(*I*)
graphite	*R*_int_ = 0.020
Detector resolution: 16.1500 pixels mm^-1^	θ_max_ = 28.7°, θ_min_ = 3.3°
ω scans	*h* = −11→12
Absorption correction: multi-scan (*CrysAlis RED*; Oxford Diffraction, 2010)	*k* = −18→18
*T*_min_ = 0.963, *T*_max_ = 0.977	*l* = −12→13
11331 measured reflections	

### Refinement

**Table:**

Refinement on *F*^2^	Secondary atom site location: difference Fourier map
Least-squares matrix: full	Hydrogen site location: inferred from neighbouring sites
*R*[*F*^2^ > 2σ(*F*^2^)] = 0.042	H atoms treated by a mixture of independent and constrained refinement
*wR*(*F*^2^) = 0.117	*w* = 1/[σ^2^(*F*_o_^2^) + (0.0567*P*)^2^ + 0.3281*P*] where *P* = (*F*_o_^2^ + 2*F*_c_^2^)/3
*S* = 1.02	(Δ/σ)_max_ = 0.001
3231 reflections	Δρ_max_ = 0.25 e Å^−^^3^
177 parameters	Δρ_min_ = −0.18 e Å^−^^3^
1 restraint	Extinction correction: *SHELXL97* (Sheldrick, 2008), Fc^*^=kFc[1+0.001xFc^2^λ^3^/sin(2θ)]^-1/4^
Primary atom site location: structure-invariant direct methods	Extinction coefficient: 0.059 (4)

### Special details

**Table:**

Geometry. All esds (except the esd in the dihedral angle between two l.s. planes) are estimated using the full covariance matrix. The cell esds are taken into account individually in the estimation of esds in distances, angles and torsion angles; correlations between esds in cell parameters are only used when they are defined by crystal symmetry. An approximate (isotropic) treatment of cell esds is used for estimating esds involving l.s. planes.
Refinement. Refinement of *F*^2^ against ALL reflections. The weighted *R*-factor *wR* and goodness of fit *S* are based on *F*^2^, conventional *R*-factors *R* are based on *F*, with *F* set to zero for negative *F*^2^. The threshold expression of *F*^2^ > σ(*F*^2^) is used only for calculating *R*-factors(gt) *etc*. and is not relevant to the choice of reflections for refinement. *R*-factors based on *F*^2^ are statistically about twice as large as those based on *F*, and *R*- factors based on ALL data will be even larger.

### Fractional atomic coordinates and isotropic or equivalent isotropic displacement parameters (Å^2^)

**Table:**

	*x*	*y*	*z*	*U*_iso_*/*U*_eq_	
O1	0.67772 (14)	0.53971 (7)	1.02990 (9)	0.0554 (3)	
O2	0.65842 (11)	0.66691 (6)	0.89536 (10)	0.0444 (2)	
O3	0.46662 (10)	0.59366 (8)	0.66899 (10)	0.0467 (3)	
O4	0.67624 (12)	0.86689 (8)	0.21951 (11)	0.0538 (3)	
H4O	0.7691 (17)	0.8749 (14)	0.2078 (19)	0.065*	
C1	0.6277 (3)	0.59825 (13)	1.13629 (15)	0.0679 (5)	
H1A	0.6325	0.5611	1.2192	0.102*	
H1B	0.5236	0.6189	1.1131	0.102*	
H1C	0.6930	0.6545	1.1487	0.102*	
C2	0.68941 (13)	0.58440 (9)	0.91453 (12)	0.0353 (3)	
C3	0.75070 (13)	0.52066 (8)	0.81331 (12)	0.0339 (3)	
C4	0.85127 (16)	0.44807 (10)	0.85257 (14)	0.0452 (3)	
H4A	0.8748	0.4357	0.9449	0.054*	
C5	0.91727 (18)	0.39370 (11)	0.75845 (17)	0.0531 (4)	
H5A	0.9850	0.3437	0.7862	0.064*	
C6	0.88503 (17)	0.41180 (10)	0.62450 (16)	0.0494 (3)	
H6A	0.9308	0.3745	0.5599	0.059*	
C7	0.78597 (15)	0.48435 (9)	0.58380 (13)	0.0404 (3)	
H7A	0.7654	0.4972	0.4913	0.048*	
C8	0.71643 (12)	0.53855 (8)	0.67704 (11)	0.0322 (2)	
C9	0.59158 (13)	0.60509 (8)	0.62820 (11)	0.0326 (2)	
C10	0.61847 (12)	0.67596 (8)	0.52514 (11)	0.0308 (2)	
C11	0.76378 (12)	0.70441 (8)	0.49875 (12)	0.0331 (3)	
H11A	0.8483	0.6784	0.5502	0.040*	
C12	0.78631 (13)	0.76950 (9)	0.39946 (12)	0.0353 (3)	
H12A	0.8855	0.7890	0.3835	0.042*	
C13	0.66243 (14)	0.80656 (9)	0.32274 (12)	0.0359 (3)	
C14	0.51685 (13)	0.78068 (9)	0.34968 (13)	0.0384 (3)	
H14A	0.4325	0.8073	0.2987	0.046*	
C15	0.49546 (13)	0.71679 (9)	0.44985 (12)	0.0352 (3)	
H15A	0.3959	0.7000	0.4684	0.042*	

### Atomic displacement parameters (Å^2^)

**Table:**

	*U*^11^	*U*^22^	*U*^33^	*U*^12^	*U*^13^	*U*^23^
O1	0.0885 (8)	0.0476 (6)	0.0317 (5)	0.0005 (5)	0.0142 (5)	0.0028 (4)
O2	0.0508 (5)	0.0381 (5)	0.0459 (5)	0.0019 (4)	0.0124 (4)	0.0004 (4)
O3	0.0334 (5)	0.0645 (6)	0.0436 (5)	0.0006 (4)	0.0112 (4)	0.0083 (4)
O4	0.0446 (5)	0.0645 (7)	0.0533 (6)	0.0042 (5)	0.0097 (5)	0.0255 (5)
C1	0.1059 (15)	0.0657 (10)	0.0344 (7)	−0.0035 (10)	0.0198 (8)	−0.0063 (7)
C2	0.0359 (6)	0.0384 (6)	0.0315 (6)	−0.0050 (5)	0.0029 (4)	0.0014 (4)
C3	0.0351 (6)	0.0321 (5)	0.0349 (6)	−0.0029 (4)	0.0046 (4)	0.0023 (4)
C4	0.0487 (7)	0.0425 (7)	0.0443 (7)	0.0041 (5)	0.0037 (6)	0.0103 (5)
C5	0.0536 (8)	0.0420 (7)	0.0647 (9)	0.0140 (6)	0.0101 (7)	0.0088 (6)
C6	0.0512 (8)	0.0415 (7)	0.0572 (9)	0.0084 (6)	0.0144 (6)	−0.0060 (6)
C7	0.0430 (7)	0.0410 (6)	0.0379 (6)	0.0008 (5)	0.0081 (5)	−0.0042 (5)
C8	0.0318 (5)	0.0317 (5)	0.0337 (6)	−0.0026 (4)	0.0054 (4)	0.0004 (4)
C9	0.0308 (5)	0.0383 (6)	0.0289 (5)	−0.0003 (4)	0.0041 (4)	−0.0033 (4)
C10	0.0287 (5)	0.0350 (6)	0.0289 (5)	0.0018 (4)	0.0036 (4)	−0.0028 (4)
C11	0.0274 (5)	0.0398 (6)	0.0317 (5)	0.0021 (4)	0.0005 (4)	−0.0015 (4)
C12	0.0286 (5)	0.0408 (6)	0.0368 (6)	−0.0018 (4)	0.0056 (4)	−0.0004 (5)
C13	0.0376 (6)	0.0374 (6)	0.0335 (6)	0.0036 (5)	0.0066 (5)	0.0019 (5)
C14	0.0305 (5)	0.0470 (7)	0.0375 (6)	0.0081 (5)	0.0015 (4)	0.0045 (5)
C15	0.0257 (5)	0.0438 (6)	0.0365 (6)	0.0019 (4)	0.0043 (4)	−0.0001 (5)

### Geometric parameters (Å, °)

**Table:**

O1—C2	1.3286 (15)	C6—C7	1.3844 (19)
O1—C1	1.4458 (18)	C6—H6A	0.9500
O2—C2	1.1991 (15)	C7—C8	1.3895 (17)
O3—C9	1.2270 (14)	C7—H7A	0.9500
O4—C13	1.3508 (15)	C8—C9	1.5013 (16)
O4—H4O	0.852 (14)	C9—C10	1.4675 (16)
C1—H1A	0.9800	C10—C11	1.3993 (15)
C1—H1B	0.9800	C10—C15	1.4009 (16)
C1—H1C	0.9800	C11—C12	1.3777 (17)
C2—C3	1.4894 (17)	C11—H11A	0.9500
C3—C4	1.3896 (18)	C12—C13	1.3930 (17)
C3—C8	1.4007 (16)	C12—H12A	0.9500
C4—C5	1.382 (2)	C13—C14	1.3932 (17)
C4—H4A	0.9500	C14—C15	1.3711 (17)
C5—C6	1.377 (2)	C14—H14A	0.9500
C5—H5A	0.9500	C15—H15A	0.9500
			
C2—O1—C1	115.42 (12)	C8—C7—H7A	119.7
C13—O4—H4O	109.8 (13)	C7—C8—C3	119.21 (11)
O1—C1—H1A	109.5	C7—C8—C9	118.48 (10)
O1—C1—H1B	109.5	C3—C8—C9	121.78 (10)
H1A—C1—H1B	109.5	O3—C9—C10	121.90 (11)
O1—C1—H1C	109.5	O3—C9—C8	118.49 (11)
H1A—C1—H1C	109.5	C10—C9—C8	119.39 (10)
H1B—C1—H1C	109.5	C11—C10—C15	118.39 (10)
O2—C2—O1	124.05 (12)	C11—C10—C9	122.18 (10)
O2—C2—C3	124.01 (11)	C15—C10—C9	119.43 (10)
O1—C2—C3	111.90 (10)	C12—C11—C10	121.12 (10)
C4—C3—C8	119.47 (11)	C12—C11—H11A	119.4
C4—C3—C2	120.49 (11)	C10—C11—H11A	119.4
C8—C3—C2	119.85 (10)	C11—C12—C13	119.43 (11)
C5—C4—C3	120.55 (13)	C11—C12—H12A	120.3
C5—C4—H4A	119.7	C13—C12—H12A	120.3
C3—C4—H4A	119.7	O4—C13—C12	122.68 (11)
C6—C5—C4	120.09 (13)	O4—C13—C14	117.13 (11)
C6—C5—H5A	120.0	C12—C13—C14	120.18 (11)
C4—C5—H5A	120.0	C15—C14—C13	119.95 (11)
C5—C6—C7	120.04 (13)	C15—C14—H14A	120.0
C5—C6—H6A	120.0	C13—C14—H14A	120.0
C7—C6—H6A	120.0	C14—C15—C10	120.87 (10)
C6—C7—C8	120.61 (12)	C14—C15—H15A	119.6
C6—C7—H7A	119.7	C10—C15—H15A	119.6
			
C1—O1—C2—O2	−1.6 (2)	C3—C8—C9—O3	−51.01 (16)
C1—O1—C2—C3	176.40 (13)	C7—C8—C9—C10	−54.18 (15)
O2—C2—C3—C4	147.15 (13)	C3—C8—C9—C10	134.26 (12)
O1—C2—C3—C4	−30.85 (16)	O3—C9—C10—C11	165.69 (12)
O2—C2—C3—C8	−27.74 (18)	C8—C9—C10—C11	−19.76 (16)
O1—C2—C3—C8	154.27 (11)	O3—C9—C10—C15	−14.46 (17)
C8—C3—C4—C5	0.0 (2)	C8—C9—C10—C15	160.09 (11)
C2—C3—C4—C5	−174.93 (12)	C15—C10—C11—C12	−1.31 (17)
C3—C4—C5—C6	0.7 (2)	C9—C10—C11—C12	178.54 (11)
C4—C5—C6—C7	−0.2 (2)	C10—C11—C12—C13	−0.95 (18)
C5—C6—C7—C8	−1.0 (2)	C11—C12—C13—O4	−176.60 (12)
C6—C7—C8—C3	1.68 (19)	C11—C12—C13—C14	2.41 (18)
C6—C7—C8—C9	−170.10 (12)	O4—C13—C14—C15	177.49 (12)
C4—C3—C8—C7	−1.16 (17)	C12—C13—C14—C15	−1.58 (19)
C2—C3—C8—C7	173.77 (11)	C13—C14—C15—C10	−0.74 (19)
C4—C3—C8—C9	170.33 (11)	C11—C10—C15—C14	2.16 (17)
C2—C3—C8—C9	−14.73 (16)	C9—C10—C15—C14	−177.69 (11)
C7—C8—C9—O3	120.55 (13)		

### Hydrogen-bond geometry (Å, °)

**Table:**

*D*—H···*A*	*D*—H	H···*A*	*D*···*A*	*D*—H···*A*
O4—H4O···O3^i^	0.85 (1)	1.88 (2)	2.7310 (14)	173.(2)
C12—H12A···O2^i^	0.95	2.50	3.4333 (15)	167

Symmetry codes: (i) *x*+1/2, −*y*+3/2, *z*−1/2.
